# Therapeutic Evidence of Human Mesenchymal Stem Cell Transplantation for Cerebral Palsy: A Meta-Analysis of Randomized Controlled Trials

**DOI:** 10.1155/2020/5701920

**Published:** 2020-07-20

**Authors:** Baocheng Xie, Minyi Chen, Runkai Hu, Weichao Han, Shaobo Ding

**Affiliations:** Affiliated Dongguan People's Hospital, Southern Medical University Dongguan, Guangdong, China

## Abstract

Cerebral palsy (CP) is a kind of movement and posture disorder syndrome in early childhood. In recent years, human mesenchymal stem cell (hMSC) transplantation has become a promising therapeutic strategy for CP. However, clinical evidence is still limited and controversial about clinical efficacy of hMSC therapy for CP. Our aim is to evaluate the efficacy and safety of hMSC transplantation for children with CP using a meta-analysis of randomized controlled trials (RCTs). We conducted a systematic literature search including Embase, PubMed, ClinicalTrials.gov, Cochrane Controlled Trials Register databases, Chinese Clinical Trial Registry, and Web of Science from building database to February 2020. We used Cochrane bias risk assessment for the included studies. The result of pooled analysis showed that hMSC therapy significantly increased gross motor function measure (GMFM) scores (standardized mean difference (SMD) = 1.10, 95%CI = 0.66‐1.53, *P* < 0.00001, high-quality evidence) and comprehensive function assessment (CFA) (SMD = 1.30, 95%CI = 0.71‐1.90, *P* < 0.0001, high-quality evidence) in children with CP, compared with the control group. In the subgroup analysis, the results showed that hMSC therapy significantly increased GMFM scores of 3, 6, and 12 months and CFA of 3, 6, and 12 months. Adverse event (AE) of upper respiratory infection, diarrhea, and constipation was not statistically significant between the two groups. This meta-analysis synthesized the primary outcomes and suggested that hMSC therapy is beneficial, effective, and safe in improving GMFM scores and CFA scores in children with CP. In addition, subgroup analysis showed that hMSC therapy has a lasting positive benefit for CP in 3, 6, and 12 months.

## 1. Introduction

Cerebral palsy (CP) is a syndrome of posture disorders and movement disorders caused by nonprogressive damage in brain development. Patients with CP are associated with sensory and perceptual impairments, cognition difficulties, and behavioral disorders, as well as secondary musculoskeletal disorders and epilepsy [1, 2]. Movement disorders in CP are often accompanied by sensory, perceptual, cognitive, communication, and behavioral disorders [2]. Although with the development of obstetrics and perinatology, the prevalence of CP is 2 and 3 per 1000 live births, CP is considered the major cause of disabilities and death of childhood. CP in children has become a very important public health issue that severely affected patients' quality of life and caused a burden on the patient's family and national financial resources [3, 4]. At present, the main treatment is to rely on orthopedic surgery, hyperbaric oxygen treatments, and neurotrophic drugs. The clinical efficacy is limited since there is no advantage of treatment for CP. Therefore, clinicians need to seek a novel therapeutic option for CP to improve quality of life and promote physical function of patients.

In recent years, stem cells transplantation was considered as a promising treatment strategy in clinical practices and various clinical trials [5–7]. Therefore, studies on stem cell therapy for cerebral palsy provide a new treatment strategy. Currently, the stem cells mainly used to treat CP are neural progenitor cells, hematopoietic stem cells, bone marrow mesenchymal stem cell (BMSC), and umbilical cord mesenchymal stem cell (UC-MSC) [8–11]. Compared with other types of stem cells, human mesenchymal stem cells (hMSCs) have the potential advantages of easy accessibility, immunosuppression, and low immunogenicity, so they are attractive and promising in treating various diseases. A trial of UC-MSC transplantation for children with CP showed that UC-MSC transplantation could significantly increase GMFM and CFA scores at 3, 6, 12, and 24 months. The study indicated that UC-MSC transplantation would be effective in improving functions for CP [12]. Another RCT also found that UC-MSC transplantation significantly improved GMFM and CFA scores without statistical significance in the incidence of AE between the two groups [13]. Increasing evidences show that hMSC transplantation has a therapeutic potential in the treatment of CP in some clinical studies. However, there is a lack of evidence-based medical evidence whether hMSC transplantation could treat CP. In this study, we sought to evaluate the efficacy and safety of hMSC transplantation therapy for CP by grading of recommendation assessment, development, and evaluation (GRADE) of RCTs.

## 2. Materials and Methods

The detailed protocol, which followed the template of Cochrane review for interventions, is registered in the PROSPERO (CRD42020171773). The preferred reporting items for systematic reviews and meta-analysis (PRISMA) were used to complete this study.

### 2.1. Literature Search

A comprehensive literature search was performed in Embase database, Cochrane Library, PubMed database, Web of Science, Chinese Clinical Trial Registry, and Clinical Trials.gov from building the database until February 2020. The MeSH and keywords search terms included the following: # (a) Cerebral palsy, CP, # (b) Human mesenchymal stem cell, hMSC, umbilical cordderived mesenchymal stem cell, mesenchymal stem cell, MSC, # (c) Randomized controlled trials.

### 2.2. Data Extraction

Two reviewers (Xie BC and Chen MY) screened the full-text content of RCTs of hMSC therapy in CP and extracted experimenter data in predesigned data extraction form. Controversial opinion was resolved by consensus by the third independent investigator (Han WC). Data extracted were key variables of study design and registration, number of eligible patients, average age of patients, therapeutic strategy, follow-up time, and primary outcome.

### 2.3. Assessment of Risk of Bias

To address the risk of bias of studies, we used the Cochrane bias risk tool to evaluate RCTs. We evaluated the research methodology one by one according to the items listed as follows: (1) adequacy of random sequence generation, (2) allocation concealment, (3) blinding of study participants, (4) incomplete outcome data reporting, (5) selective outcome depiction, and (6) other potential sources of bias.

### 2.4. Outcome Measures

(1) The primary efficacy outcomes are as follows: gross motor function measure (GMFM) scores of 3, 6, and 12 months and comprehensive function assessment (CFA) of 3, 6, and 12 months. (2) The primary safety outcomes are as follows: adverse event (AE) of upper respiratory infection, diarrhea, and constipation.

### 2.5. Quality of Evidence

We use the GRADE methodology to assess the quality of evidence of pooled outcome indicators. We mainly use GRADE pro software to evaluate the outcome indicators with the bias, inconsistency, discontinuity, imprecision, and risk of publication bias and then evaluate the quality of evidence as very low, low, medium, or high.

### 2.6. Inclusion and Exclusion Criteria

The inclusion criteria of our study included (1) RCTs; (2) eligibility criteria for participants included a diagnosis of CP; (3) hMSC group treated with hMSC therapy and control group treated with normal saline or rehabilitation therapy; and (4) follow-up of at least 3 months. We excluded studies that met the following criteria: (1) nonrandomized trials; (2) republished studies; (3) ongoing RCTs and retraction study; (4) less than 3 months of follow-up; (5) review and meta-analysis; and (6) letters, case reports, cross-sectional studies, cohort studies, purely experimental design scheme researches, and articles without reporting outcomes of primary data articles.

### 2.7. Data Synthesis and Analysis

The statistical interpretation of data was performed using Review Manager 5.3 software and STATA 13.0 software. Dichotomous data were analyzed using risk ratio (RR) with 95% confidence intervals (CIs). Continuous data were presented as standardized mean difference (SMD) with 95% CI. Heterogeneity among RCTs for each outcome was calculated by means of the *χ*^2^ test and *I*^2^ statistic, where *I*^2^ < 25% represents slight inconsistency, *I*^2^ between 25% and 50% with a medium heterogeneity. If *I*^2^ > 50%, the study had a severe heterogeneity; we conservatively used random-effects models to estimate the pooled outcomes to reduce the heterogeneity of studies. If not, pooled outcomes were estimated with a fixed effects model with RR and 95% CI. We performed sensitivity analyses to evaluate the robustness of the model and the impact of selected measures of study characteristics for the primary study outcomes. We performed the subgroup analyses to explore potential effects of GMFM scores of 3, 6, and 12 months and CFA of 3, 6, and 12 months.

## 3. Results

### 3.1. Data Selection

**Figure 1:**
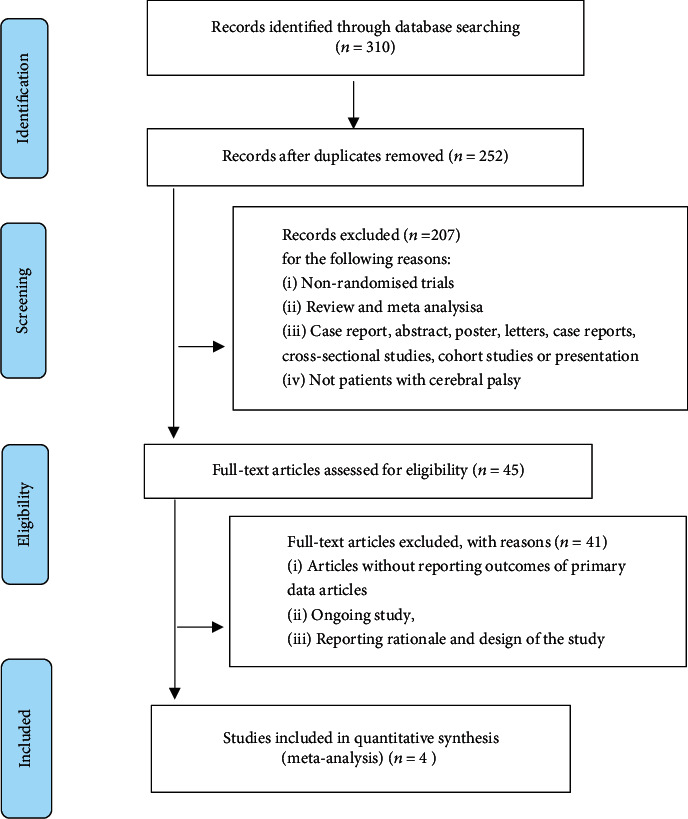
Flow diagram and strategy of this meta-analysis.

Our systematic search identified 310 citations published from building the database until February 2020. A total of 58 duplicated studies were excluded in NoteExpress. Then, after reading the titles and abstracts of the literature, we further excluded 207 studies with the following reasons: (a) nonrandomized trials; (b) review and meta-analysis; (c) case report, abstract, poster, letters, case reports, cross-sectional studies, cohort studies, or presentation; and (d) not patients with CP. Next, we excluded 41 studies of articles without reporting outcomes of primary data articles, ongoing study, and the study reporting rationale and design after reading the full text of the literature. Finally, we included 4 studies on hMSC transplantation for CP in this meta-analysis (Figure 1).

### 3.2. Characteristics of Included Studies

Four studies of 189 participants were included in this analysis. The hMSC group was treated with hMSC therapy and the control group was treated with normal saline. The clinical trial registration numbers of the three RCTs were ChiCTR1800016554, CHiCTR-TRC-12002568, and NCT01929434. The stem cell therapy used in the three RCTs was hUC-MSCs; the other stem cell used in the RCT was BMSC. The amount of hMSC transplants in RCTs of Huang et al. [12] and Gu et al. [13] and were 5 × 10^7^ and (5.00 ± 0.50) × 10^7^. The dosage of hMSC transplants in the other two studies was 1 × 10^6^/kg and 1 × 10^7^. The primary efficacy outcomes in RCTs were GMFM scores of 3, 6, and 12 months and CFA of 3, 6, and 12 months. The primary safety outcomes in RCTs were AE of upper respiratory infection, diarrhea, constipation, and fever (Table 1).

### 3.3. Quality Assessment

**Figure 2:**
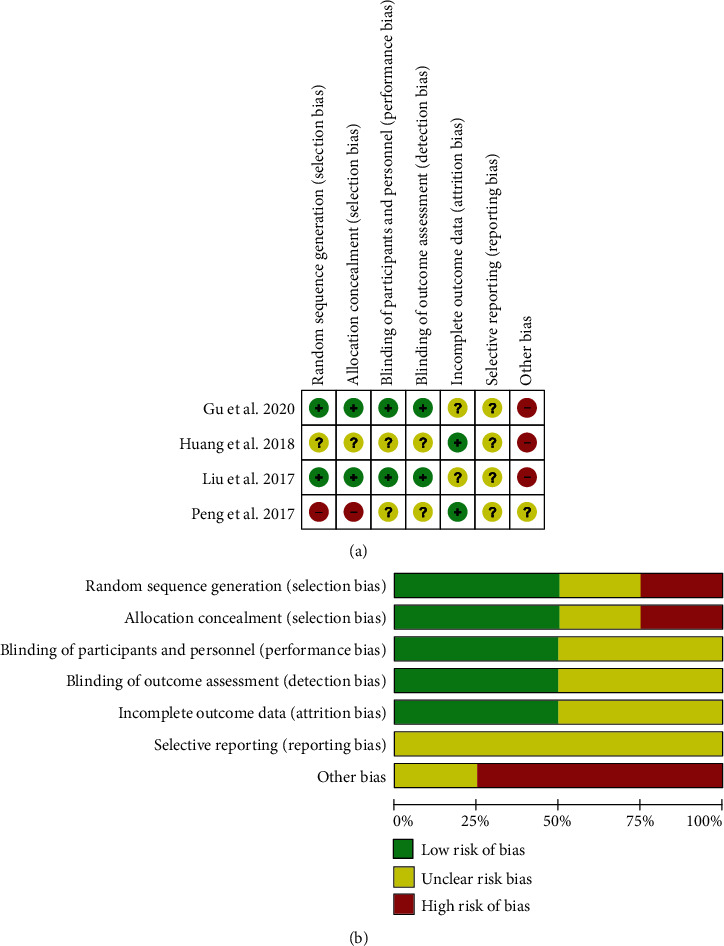
The quality assessment of each study according to the Cochrane collaboration manual. (a) Detailed analysis one by one of the risk of bias summary of included studies. (b) The risk bias graph shows a summary of the quality of each study.

The RCTs of Liu et al. [14] and Gu et al. [13] were assigned to two groups according to the randomization table. We evaluated them as “low risk” studies in selection bias. RCTs of Huang et al. and Peng et al. [15] did not report randomized methods and were assessed of “unclear risk” and “high risk” in selection bias. After randomization, the study processes of Liu et al. [14] and Gu et al. [13] were blinded to the patient groups, participant surgeons, coordinators, and the investigators. We evaluated them as “low risk” in selection bias, performance bias, and detection bias. The study of Huang et al. reported that the patients and their families were blinded. But, we did not find out whether the study was reported blind to the investigators and participant surgeons; we evaluated it as “unclear risk” in selection bias, performance bias, and detection bias. The studies of Gu et al. and Liu et al. [14] reported that one patient and two patients in the hMSC group were lost to follow-up. We evaluated them as “unclear risk” in attrition bias. The results of the studies showed low correlation between the impact of patients' lifestyle and privacy, and we considered that reporting bias with the low possibility and evaluated them as “unclear risk” in reporting bias (Figure 2).

### 3.4. Quality of Evidence

We used the GRADE methodology to assess quality of evidence. We evaluated that hMSC therapy significantly increased GMFM scores and CFA score with high-quality evidence. We evaluated that AE of upper respiratory infection, diarrhea, and constipation was not statistically significant with moderate-quality evidence, between the hMSC therapy group and the control group (Table 2).

### 3.5. GMFM Scores

**Figure 3:**
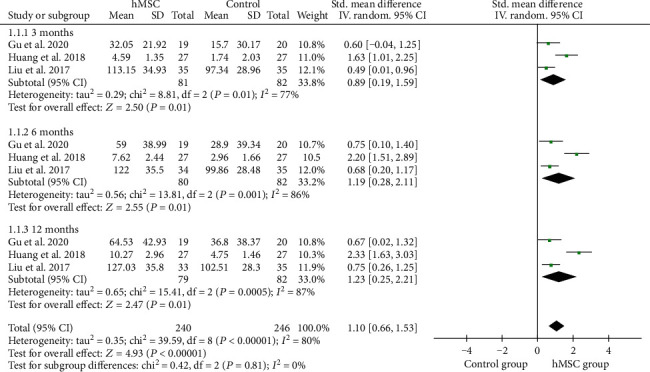
Forest plot of the meta-analysis with GMFM scores between the hMSC therapy and control groups.

GMFM scores were reported in 3 studies of 81 patients with hMSC therapy and 82 patients in the control group. We used a random-effects model after heterogeneity analysis (*I*^2^ = 80 > 50%). Pooled analysis showed that hMSC therapy significantly increased GMFM scores (SMD = 1.10, 95%CI = 0.66‐1.53, *P* < 0.00001, high-quality evidence) (Figure 3, Table 2), compared with the control group. Subgroup analysis with random-effects model showed that hMSC therapy significantly increased GMFM scores in 3 months (SMD = 0.89, 95%CI = 0.19‐1.59, *P* = 0.01), 6 months (SMD = 1.19, 95%CI = 0.28‐2.11, *P* = 0.01), and 12 months (SMD = 1.23, 95%CI = 0.25‐2.21, *P* = 0.01), compared with the control group in children with CP (Figure 3).

### 3.6. CFA Scores

**Figure 4:**
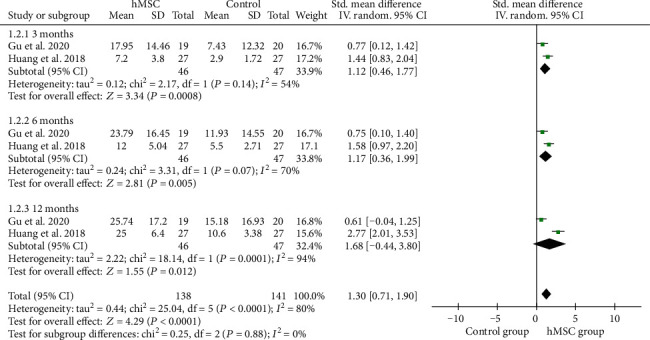
Forest plot of the meta-analysis with CFA scores between the hMSC therapy and control groups.

CFA scores were reported in 2 RCTs of 46 patients treated with hMSC therapy and 47 patients in the control group. A random-effects model was used to analyze after heterogeneity analysis (*I*^2^ = 80%). Pooled analysis indicated that hMSC therapy significantly improved CFA scores (SMD = 1.30, 95%CI = 0.71‐1.90, *P* < 0.0001, high-quality evidence) (Figure 4, Table 2), compared with the control group. Subgroup analysis with random-effects model showed that hMSC therapy significantly increased CFA scores in 3 months (SMD = 1.12, 95%CI = 0.46‐1.77, *P* = 0.0008) and 6 months (SMD = 1.17, 95%CI = 0.36‐1.99, *P* = 0.005) in children with CP (Figure 4).

### 3.7. Adverse Event (AE)

In order to explore the safety of hMSC therapy, we conducted a meta-analysis of AE. Pooled analysis indicated that AE of upper respiratory infection (RR = 0.80, 95%CI = 0.34‐1.87, *P* = 0.60, moderate-quality evidence), diarrhea (RR = 0.81, 95%CI = 0.42‐1.57, *P* = 0.53, moderate-quality evidence), and constipation (RR = 0.59, 95%CI = 0.13‐2.62, *P* = 0.59, moderate-quality evidence) was not statistically significant between the hMSC therapy group and the control group (Tables 2 and 3). There was no statistical significance in other adverse events, such as fever, vomiting, anorexia, and urticaria in the studies. There were two studies [14, 15] which had low intracranial pressure after lumbar puncture in the hMSC transplantation group, including mild dizziness and headache, nausea, and vomiting. But, the symptoms of the children were relieved and disappeared when the patients lay on the bed without pillows and were treated with intravenous drip of saline.

## 4. Discussion

### 4.1. Primary Efficacy Outcomes

GMFM scores are useful and important as outcome evaluation results to evaluate changes in gross motor function for CP after interventions. This is crucial to determine effectiveness and benefit of interventional therapy by measuring the change of gross motor skill acquisition in children with CP. Children's measure gross motor function is commonly evaluated by rehabilitation specialists using GMFM scores. GMFM scored items consist of 5 parts: lying and rolling (17 items); walking, running, and jumping (24 items); sitting (20 items); climbing and kneeling (14 items); and standing (13 items). The items are scored in a four-point order (cannot initiate item, 0; initiates item, 1; partially completes item, 2; and completes item independently, 3) [16]. Higher scores in GMFM scores indicate better capacity and favourable prognosis in children with CP. The study of Wang et al. recruited 16 patients with CP and received UCMSC transplantation and the result showed that GMFM scores had significant improvement at the end of the first and sixth months after UCMSC transplantation [17]. Another study was of 52 patients with CP who received BMSC transplantation. The gross motor function was evaluated using GMFM scores in 1, 6, and 18 months. The result showed that BMMSC transplantation could significantly increase the GMFM scores at 6 months and 18 months of patients with CP, compared with the baseline value [18]. To further provide reliable evidence and high-quality evidence, we included three RCTs of hMSC therapy in CP and pooled results showed that hMSC therapy significantly increased GMFM scores in children with CP, compared with the control group. Moreover, we performed a subgroup analysis of GMFM scores of 3, 6, and 12 months. The result of subgroup analysis showed that hMSC therapy significantly increased GMFM scores in 3, 6, and 12 months (*P* = 0.01). We evaluated the indicators of GMFM scores with high-quality evidence using GRADE including inconsistency, risk of bias, indirectness, publication bias, and imprecision.

CFA is mainly used to evaluate function improvement and therapeutic effect of patients with CP. The RCTs of Gu et al. [13] and Huang et al. reported the changes of CFA in patients with CP after hMSC therapy. The results of both studies have shown that hMSC therapy could significantly improve CFA in patients with CP. In our study, we combined the data of RCTs with a total of 46 patients treated in hMSC therapy. Pooled analysis indicated that hMSC therapy significantly improved CFA scores, compared with the control group. Furthermore, we conducted a subgroup analysis on CFA scores. Subgroup analysis showed that hMSC therapy significantly increased CFA scores in 3 months (*P* = 0.0008) and 6 months (*P* = 0.005), compared with the control group in children with CP. According to GRADE, we consider that hMSC therapy for CP can improve the comprehensive function of patients with high-quality evidence. Fine motor function measure (FMFM) was also used to evaluate the therapeutic effect of cell therapy, although the study of Wang et al. [17] found that it was not statistically significant in UCMSC therapy for CP at the end of the first and sixth months. The scores of FMFM scores in the BMMSC group were all higher than those of the bone marrow mononuclear cell and the control groups at 3, 6, and 12 months after cell therapy for CP [14]. Salivation is a common symptom of patients with cerebral palsy, which seriously affects the health status of patients. The study found that UCMSC transplantation could significantly improve drooling severity and frequency scale in CP.

### 4.2. Primary Safety Outcomes

MSCs are attractive and promising because of their low immunogenicity, easy accessibility, and immunosuppressive potential in autologous transplantation [19, 20]. However, the safety of stem cell therapy remains a top priority. The studies showed that the quality of the hMSC relies on the separation conditions and cell culture techniques as well as the age, genetic traits, and different donor's medical history [21–23]. The quality of the hMSC is closely related to adverse events. Therefore, the safety of MSC transplantation involves many factors; it is necessary to evaluate the safety of MSC therapy for CP. We included 4 RCTs on hMSC therapy for CP. The RCT of Gu et al. [13] reported the incidence of upper respiratory infection (52.63%), diarrhea (31.58%), fever (36.84%), and constipation (5.26%) in the hMSC group and upper respiratory infection (70.00%), diarrhea (45.00%), fever (15.00%), and constipation (15.00%) in the control group. The RCT of Huang et al. [12] also reported the incidence of upper respiratory infection (33.33%), diarrhea (18.52%), and constipation (7.41%) in the hMSC group and upper respiratory infection (29.62%), diarrhea (18.52%), and constipation (7.41%) in the control group. Therefore, in order to evaluate the safety of hMSC therapy for CP, we conducted a meta-analysis for AE. Pooled analysis indicated that AE of upper respiratory infection (*P* = 0.60, moderate-quality evidence), diarrhea (*P* = 0.53, moderate-quality evidence), and constipation (*P* = 0.59, moderate-quality evidence) was not statistically significant between the two groups. There was no statistical significance in other adverse events, such as fever, vomiting, anorexia, and urticaria in the studies. Serious adverse events were not observed in the included studies. However, there were two studies [14, 15] which had low intracranial pressure after lumbar puncture in the hMSC transplantation group. The symptoms of the children were relieved and disappeared when the patients lay in bed without pillows and were treated with intravenous drip of saline. The common adverse effect of hMSC transplantation by lumbar puncture is low intracranial pressure, which should be noted. The reasons for the low cranial pressure after lumbar puncture may be as follows: (1) most children have high muscle tension in their extremities, and the low cranial pressure is easy to occur after operation; (2) slender body, poor nutritional status; (3) poor cooperation of children during lumbar puncture hMSC transplantation, resulting in more puncture times; and (4) the degree of crying in the operation of children is heavier, resulting in a rapid outflow of cerebrospinal fluid. Therefore, after hMSC transplantation by lumbar puncture, targeted measures should be taken before, during, and after the operation to reduce the incidence of adverse reactions. (1) Before the operation for children and patients with involuntary exercise, the operation should be performed under sedation and hypnosis as far as possible, so as to avoid the children's crying and high limb muscle tension. (2) The lumbar puncture needle with fine caliber should be used during the operation and should reduce the number of puncture as far as possible and avoid multiple puncture of the same site in a short period of time. (3) The patients should lie down and rest after the operation and avoid raising his head and standing up. (4) Patients could take appropriate amount of normal saline according to the doctor's advice after surgery.

### 4.3. Limitations and Critical Considerations

We evaluated and analyzed the heterogeneity of included outcomes and found that there was a high heterogeneity in GMFM scores. Sensitivity analysis shows that the RCT of Huang et al. [12] resulted in high heterogeneity. In our analysis of this study, we found that the main reason for the high heterogeneity was that GMFM scores were reported in the form of the difference between the final score and the baseline data. If we exclude this study, heterogeneity will return to *I*^2^ = 0% and the pooled results are consistent with the previous trend. In addition, we analyzed the sensitivity of GMFM scores using the Galbraith plot. The results were credible with no substantial change in the GMFM score. But, the small number of studies limited the analysis of publication bias in this study.

## 5. Conclusions

In conclusion, this meta-analysis synthesized the primary outcomes which suggested that hMSC therapy was safe and more effective in improving GMFM and CFA in children with CP. Apparently, the findings provide a novel therapeutic strategy for patients with CP. However, what are the optimal dose, frequency, timing, and routes of MSC transplantation in different phases of CP? These important and challenging clinical questions need more RCTs to be addressed urgently.

## Figures and Tables

**Table 1:** Characteristics of included articles.

Study	Registered number	Design	Participants	Intervention		Dosage	Outcomes
				hMSC group	Control group		
Gu et al. [13]	ChiCTR1800016554	RCT	40	hMSC therapy	Normal saline	(5.00 ± 0.50) × 10^7^	GMFM, CFA, AE
Huang et al. [12]	NA	RCT	54	hMSC therapy	Normal saline	5 × 10^7^	GMFM, CFA, AE
Liu et al. [14]	CHiCTR-TRC-12002568	RCT	70	hMSC therapy	Rehabilitation therapy	1 × 10^6^/kg	GMFM, FMFM, AE
Peng et al. [15]	NCT01929434	RCT	25	hMSC therapy	Nonintervention therapy	1 × 10^7^	SF, AE

Note: RCT: randomized controlled trial; hMSC: human mesenchymal stem cell; GMFM: gross motor function measure; CFA: comprehensive function assessment; AE: adverse event; FMFM: fine motor function measure; SF: drooling severity and frequency scale.

**Table 2:** GRADE summary of human mesenchymal stem cell transplantation for cerebral palsy.

Outcome	Absolute effect estimates (95% CI)		Relative effect (95% CI)	Participants (studies)	Evidence (GRADE)^†^	Comments
	Control	hMSC therapy				
GMFM	—	SMD 1.1 higher (0.66 to 1.53 higher)	—	163 (3)	**⊕⊕⊕⊕** High	hMSC therapy has important benefit in increasing GMFM.
CFA	—	SMD 1.3 higher (0.71 to 1.90 higher)	—	96 (3)	**⊕⊕⊕⊕** Moderate	hMSC therapy has important benefit in increasing CFA.
Upper respiratory infection	468 per 1000	417 per 1000 (267 to 651) Difference: 51 fewer per 1000 (201 to 183)	RR 0.89 (0.57 to 1.39)	93 (2)	**⊕⊕⊕○** Moderate	hMSC therapy did not increase AE of upper respiratory infection.
Diarrhea	298 per 1000	241 per 1000 (125 to 468) Difference: 57 fewer per 1000 (173 to 170)	RR 0.81 (0.42 to 1.57)	93 (2)	**⊕⊕⊕○** Moderate	hMSC therapy did not increase AE of diarrhea.
Constipation	106 per 1000	65 per 1000 (16 to 260) Difference: 41 fewer per 1000 (90 to 153)	RR 0.61 (0.15 to 2.44)	93 (2)	**⊕⊕⊕○** Moderate	hMSC therapy did not increase AE of constipation.

^†^High: we are confident that the true effect of outcomes lies close to the estimate of the effect. Moderate: we are moderately confident that the assessed effects and the true effect are likely to be close to the assessed effects. CI: confidence interval; RR: risk ratio; AE: adverse event; SMD: standardized mean difference.

**Table 3:** Adverse event analysis between the hMSC therapy group and the control group.

AE	Study	RR and 95% CI	*P*
Upper respiratory infection	2 [12, 13]	RR (0.80), 95% CI (0.34-1.87)	0.60
Diarrhea	2 [12, 13]	RR (0.81), 95% CI (0.42-1.57)	0.53
Constipation	2 [12, 13]	RR (0.59), 95% CI (0.13-2.62)	0.59

## Supplementary Materials

Supplementary Materials Appendix S1: the PRISMA Checklist of this meta-analysis.
